# The Superficial Dermis May Initiate Keloid Formation: Histological Analysis of the Keloid Dermis at Different Depths

**DOI:** 10.3389/fphys.2017.00885

**Published:** 2017-11-07

**Authors:** Hu Jiao, Tiran Zhang, Jincai Fan, Ran Xiao

**Affiliations:** ^1^The Research Center of Plastic Surgery Hospital, Chinese Academy of Medical Sciences & Peking Union Medical College, Beijing, China; ^2^Scar Plastic Department of Plastic Surgery Hospital, Chinese Academy of Medical Sciences & Peking Union Medical College, Beijing, China

**Keywords:** keloid, histology, fibroblast, lymphocyte, collagen

## Abstract

Several studies have reported on certain aspects of the characteristics of different sites within a keloid lesion, but detailed studies on the keloid dermis at different depths within a keloid lesion are scarce. The aim of this study was to investigate the histology of the keloid dermis at different depths. This study included 19 keloid tissue samples that were collected from 19 patients and 19 normal skin samples, which were harvested from subjects without keloids or hypertrophic scar. Samples were studied by light microscopy using routine hematoxylin and eosin histochemical staining, and immunohistochemistry to detect CD20-positive B-lymphocytes and CD3-positive T-lymphocytes. Sirius Red histochemical staining was used to determine the type of collagen in keloid tissue and normal skin samples. The migratory properties of fibroblasts within the keloid dermis at different depths was compared, using an *in vitro* migration assay. The findings of this study showed that although the papillary and reticular dermis could be clearly distinguished in normal skin, three tissue layers were identified in the keloid dermis. The superficial dermis of keloid was characterized by active fibroblasts and lymphocytes; the middle dermis contained dense extracellular matrix (ECM) with large numbers fibroblasts, and the deep dermis was poorly cellular and characterized by hyalinized collagen bundles. In the keloid samples, from the superficial to the deep dermis, type I collagen increased and type III collagen decreased, and fibroblasts from the superficial dermis of the keloid were found to migrate more rapidly. In conclusion, the findings of this study showed that different depths within the keloid dermis displayed different biological features. The superficial dermis may initiate keloid formation, in which layer intralesional injection of pharmaceuticals and other treatments should be performed for keloid.

## Introduction

Keloid is a fibrotic skin condition that typically results from abnormal wound healing. Keloid is characterized by the deposition of an excess of extracellular matrix (ECM). Unlike normal or hypertrophic scars, keloids do not regress with time, but persist indefinitely and extend beyond the original wound margin to invade the surrounding normal skin tissue (Trace et al., 2016). Keloids are not only aesthetically displeasing, but they can also be both painful and functionally disabling, causing physical and psychological distress for patients (Bayat et al., 2003; Brown et al., 2008). There are several treatment approaches for keloid that have been described, including silicone dressings, pressure dressings, onion extract, corticosteroids, 5-fluorouracil, bleomycin, cryosurgery, laser therapy, and radiotherapy (Kelly, 2009; Tziotzios et al., 2012). Intralesional injection of corticosteroids alone or in combination with other therapeutic agents is the first-line treatment for keloid (Yedomon et al., 2012), but several studies have reported variable treatment efficacy (50–100%) and keloid recurrence (9–50%) (Tziotzios et al., 2012). These varied results may result from different injection protocols, including injections into the superficial or deep dermis or the center or the margin of the lesion, but this remains unclear.

Although numerous studies on keloids have been performed since the initial description by Alibert in 1806, there have been many contradictory reports in the literature. Findings from the examination of biopsies from different sites of keloid lesions may have come to different conclusions. For example, several studies have reported a variety of different biological features in the center or margin of the keloid lesion. It has been reported that fibroblasts from the keloid margin exhibit an increased proliferation rate and produce more type I and type III collagen compared with fibroblasts from intralesional sites (Syed et al., 2011). Cell morphology and fatty acid content were also different between the peripheral and central sites of the keloid (Louw et al., 1997). Protein profiling analysis has shown that mitochondrial-associated proteins were upregulated in the margin of the keloid when compared with in the center (Javad and Day, 2012). Also, the depth of the dermis included in the keloid specimen may also contribute to the conflicting findings from keloid studies. The specific immunophenotyping of the papillary dermis and the reticular dermis has been found in keloids (Bagabir et al., 2012). Unique gene expression patterns were reported in the deeper part and the superficial part of the keloid center (Seifert et al., 2008). However, in previous studies, the histological characteristics at different depths within the keloid dermis have not been specially studied.

In the current study, we investigated the histology and biology of keloid lesion at different depths, focusing on the cellular composition and collagen composition and the biological behavior of the fibroblasts at the different levels within the keloid dermis. To define the histopathology of the different keloid dermis depths would be helpful to obtain insight into the pathogenesis and the efficient treatment layer of keloids.

## Materials and methods

### Patients and samples

Nineteen keloid skin specimens were surgically excised from patients, seven men and 12 women (mean age 29.4 years; range 19–59 years). Patients fulfilled the currently accepted criteria for keloid, defined as the presence of typical skin lesions confirmed by two plastic surgeons. None of the patients included in the study had treatment within the previous 6 months. All keloid lesions caused pain or itching or displayed redness. The duration of the skin keloids ranged from 1.5 to 5 years. Keloids were located on the anterior chest wall (six), shoulder (two), chin (one), ear (seven), arm (one) or abdomen (two), and resulted from surgery (two), acne (two), vaccination (two), trauma (two), piercing (six), or were spontaneous (five).

Nineteen normal skin specimens were obtained from age-matched and sex-matched healthy control subjects who underwent surgical procedures for cosmetic or other reasons, of which eight biopsies were from periauricular skin, five were from the anterior chest wall, four were from the abdomen, one was from the shoulder and one was from the arm. Healthy control subjects had neither keloids nor hypertrophic scars. All patients and healthy control subjects were Asian.

This study was carried out in accordance with the recommendations of the Plastic Surgery Hospital Research Guidelines. All subjects gave written informed consent in accordance with the Declaration of Helsinki. The study protocol was approved by the Ethical Committee of the Plastic Surgery Hospital, Chinese Academy of Medical Sciences and Peking Union Medical College.

### Hematoxylin and eosin (H&E) histochemical staining

Excised keloids were fixed in 10% formalin for 24 h and dehydrated by standard histological procedures (Santucci et al., 2001). Excision samples were sectioned through the center of the lesion, embedded in paraffin wax, cut on a microtome (5 μm sections) to obtain sections perpendicular to the skin surface, and thin sections were mounted on glass slides. The slides were deparaffinized and hydrated using normal procedures and stained with hematoxylin and eosin (H&E). The slides were viewed using standard light microscopy.

### Immunohistochemistry

Immunohistochemical staining was conducted as previously described (Bagabir et al., 2012). Briefly, antigen was retrieved by pressure cooking for 3 min in citrate buffer (pH = 6.0). Endogenous peroxidase was blocked in 0.3% hydrogen peroxide in methanol for 30 min, and nonspecific antibody binding was blocked with 1% bovine serum albumen (BSA). Mouse monoclonal antibodies directed against CD3 and CD20 (Zhongshan, Beijing, China) were used as primary antibodies and incubated overnight at 4°C. Horseradish peroxidase-conjugated rabbit anti-mouse IgG was incubated for 45 min at room temperature as the secondary antibody and was purchased from Abcam (Hong Kong, China). Staining was achieved using a diaminobenzidine (DAB) staining kit (Zhongshan, Beijing, China) at room temperature for 5 min. Sections were counterstained with hematoxylin.

### Sirius red staining for collagen

For Sirius Red staining, the slides were deparaffinized and hydrated as in the case of H&E staining. Then the sections were immersed in Sirius Red solution (0.1% Sirius Red in saturated picric acid, pH 2.0) for 1 h, rinsed briefly in 0.01 M HCl solution and then in water for 1 min, counterstained with hematoxylin for 1 min, differentiated in acid alcohol solution, rehydrated and mounted. Slides were visualized under polarized light microscopy, and photomicrographs were taken with identical exposure settings for all sections, as previous described (Meruane et al., 2012; Wang et al., 2017).

### Fibroblasts migration

To investigate the migration of fibroblasts from keloid dermis at different depths, tissue culture was carried out. The keloid dermis was cut into a strip that contained the superficial dermis (SD), mid-dermis (MD), and deep dermis (DD). The strip of skin tissue containing the keloid dermis was placed horizontally onto the bottom of a tissue culture dish and covered with a sterilized glass coverslip. Tissues were maintained in Dulbecco's modified Eagle's medium (DMEM), supplemented with 10% fetal bovine serum (FBS) and incubated in a carbon dioxide incubator at 37°C. After 7 days, fibroblast migration was observed under microscopy and the migration distance (the perpendicular distance between the farthest fibroblast and the border of dermis strip) was measured.

### Statistical analysis

Statistical analysis was performed using SPSS version 16.0 (SPSS, Chicago, USA). Data were presented as the mean ± SD (standard deviation). Data were analyzed using Fisher's Least Significant Difference test to compare data between individual experimental groups. The level of statistical significance was *P* < 0.05.

## Results

### Histology of normal skin

The histology of keloid skin tissues and normal skin were viewed by light microscopy following routine hematoxylin and eosin (H&E) histochemical staining. In the normal skin, two layers of the superficial papillary dermis and the deeper, thicker reticular dermis were clearly distinguished (data not shown). In the dermis of the normal skin, the ECM was less compact, with fine, loosely and irregularly arranged collagen bundles and few cells. Although the thickness of the dermis from different skin biopsies was different, they showed similar histology.

### Histology of the keloid dermis at different depths

**Figure 1:**
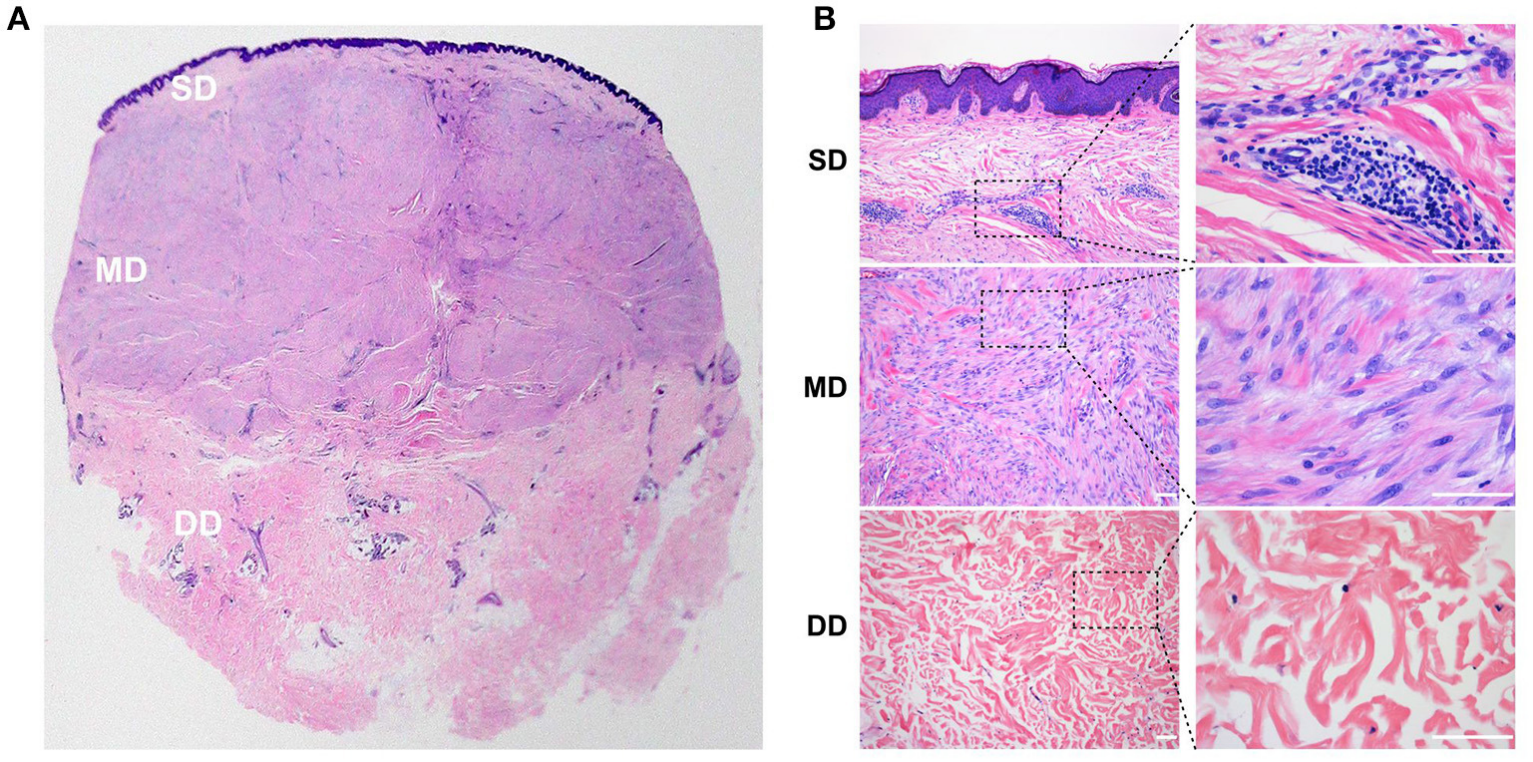
Histology of the keloid dermis at different depths. **(A,B)** In the SD, a heavy infiltrate of fibroblasts and lymphocytes was found, and collagen bundles were fine and organized parallel to each other. **(A,B)** In the MD, compact collagen bundles and abundant fibroblasts were found. **(A,B)** Atrophic and degenerate cells, as well as hyalinized collagen fibers were found in the DD. SD, superficial dermis; MD, middle dermis; DD, deep dermis. Scale bar = 100 μm.

The dermis in the keloid skin showed distinct histological features at different depths, with three tissue layers identified. The superficial dermis (SD), directly beneath the epidermis, was the thinnest tissue layer (643 ± 380 μm) and was morphologically similar to the granulation tissue observed in normal wound healing. In this layer, we found a heavy infiltrate of cells, including spindle-shaped fibroblasts and lymphocytes. The collagen bundles in the superficial dermis of the keloid were fine and organized parallel to each other (Figure 1).

The mid-dermis (MD) of the keloid was the thickest layer accounting for more than half of the thickness of the lesion, with compact collagen fibers and the most prominent fibroblast infiltration (Figure 1). Different histological characteristics were also evident from the superficial to deep regions in this layer. For example, in the superficial zone, the nuclei of the spindle-shaped cells were large and oval with prominent nucleoli, suggesting these cells were activated fibroblasts. However, the cell nuclei in the deep zone were small and exhibited pyknosis, suggesting that these cells were static fibrocytes. Collagen fibers were thick and hyalinized fibers were found in the MD.

The third layer in the keloid dermis was the deep dermis (DD), which featured prominent degeneration and necrosis of dermal cells (Figure 1). Atrophic and degenerate cells, the basophilic nuclear remnants of which scattered in the ECM, were observed here. Collagen bundles were abnormally thick as well as randomly and loosely organized, and more hyalinized collagen fibers were found in this layer.

Though keloid lesions were harvested from different parts of the body, there were no obvious differences in their histology. In our study, keloid duration was also not related to the histology, which may have resulted from small sample size and short duration of disease.

### Lymphocyte infiltration in the keloid dermis at different depths

**Figure 2:**
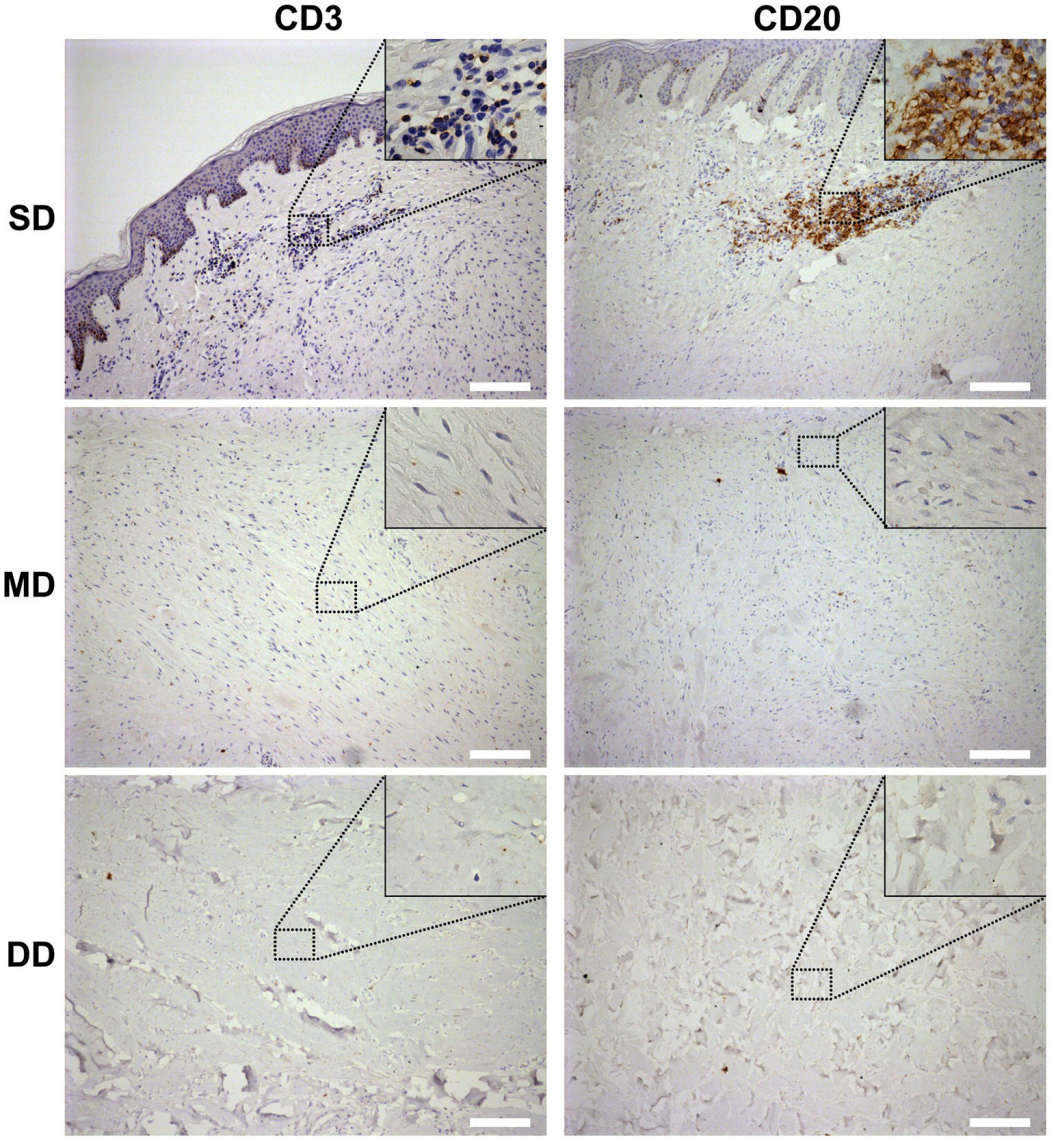
Lymphocyte infiltration in the keloid dermis at different depths. CD3^+^ T-lymphocytes and CD20^+^ B-lymphocytes in keloid was detected by immunohistochemistry staining. SD, the superficial dermis of keloids; MD, the middle dermis of keloids; DD, the deep dermis of keloids. Scale bar = 200 μm.

To confirm whether those small round cells with large nucleus infiltrated in the SD of the keloid the markers of T-lymphocytes and B-lymphocytes, CD3 and CD20 were examined by immunohistochemistry staining (Figure 2). Results showed the small round cells were positive for CD3 or CD20. CD3^+^ T-lymphocytes were mainly located in close association with vessels, whereas CD20^+^ B-lymphocytes formed large and compact aggregates in the SD of the keloid. However, few of CD3- positive T-lymphocytes or CD20-positive B-lymphocytes were found in the MD or DD of the keloid, or in the dermis of normal skin.

### Histology of the keloid dermis at different sites

**Figure 3:**
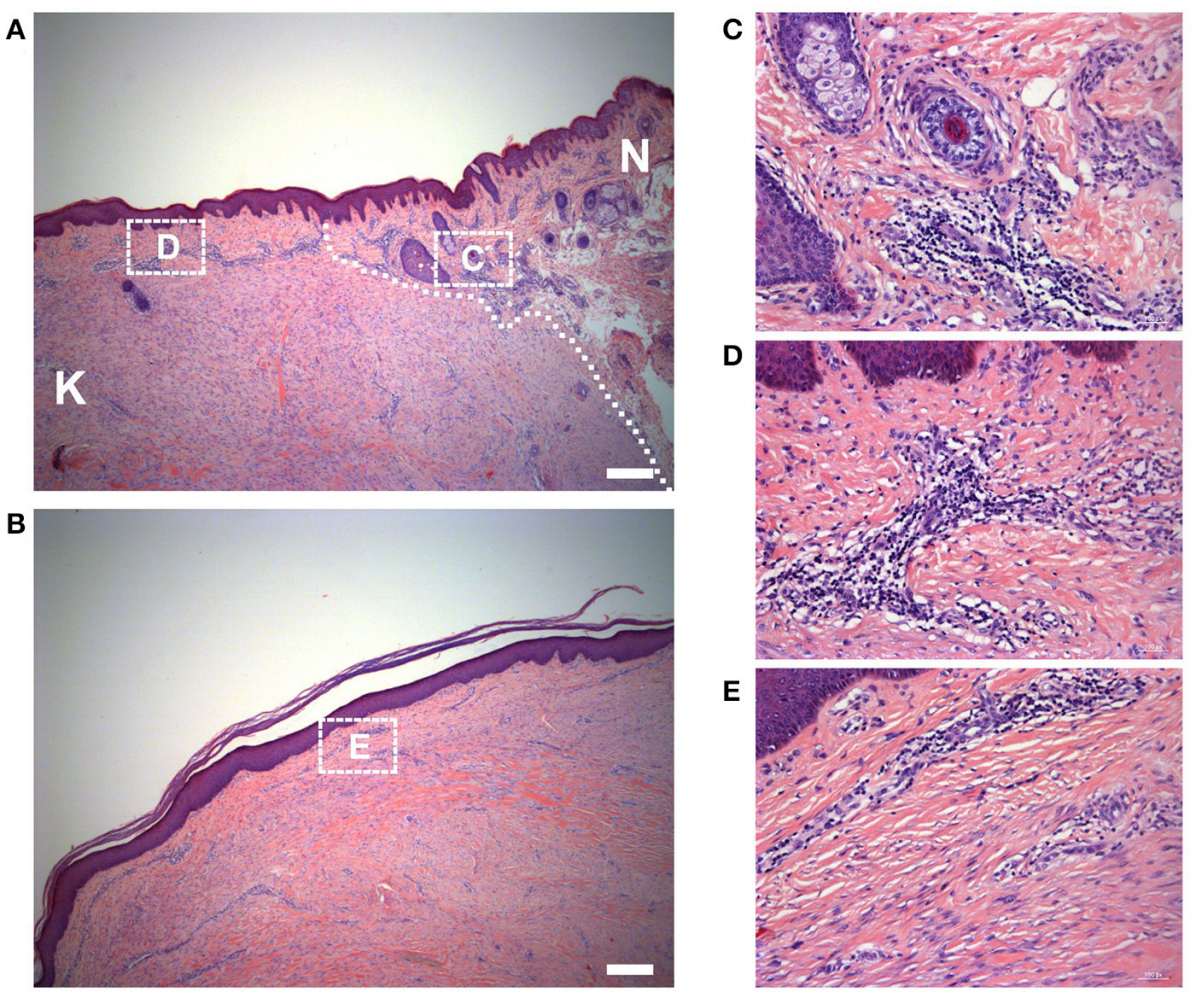
Histology of the keloid dermis at different sites. **(A)** The boundary and **(B)** the center of keloid lesion were observed. The interface of lesion and normal skin was identifiable microscopically (white dotted line indicated) and **(C)** the macroscopically normal-appearing skin surrounding the keloid lesion exhibited a prominent infiltration of lymphocytes. **(B,E)** In the center of the keloid lesion, the infiltration of lymphocytes was less than it in the margin of the lesion **(D)**. K, keloid; N, the macroscopically normal-appearing skin surrounding the keloid lesion. Scale bar = 200 μm.

We also observed the surrounding normal skin tissue, the margin and the center of the keloid tissue. The interface between the keloid tissue and the surrounding normal skin tissue was identifiable microscopically. The ECM was loose and skin appendages were present in normal skin; the ECM was dense and the skin appendages were absent in keloids (Figure 3A). In addition, the macroscopically normal skin surrounding the keloid lesion exhibited a prominent infiltration of lymphocytes (Figure 3C). Also, the infiltration of lymphocytes in the margin of the keloid lesion (Figure 3D) was greater than in the center of the lesion (Figures 3B,E).

### Collagen composition in keloid lesions and normal skin

**Figure 4:**
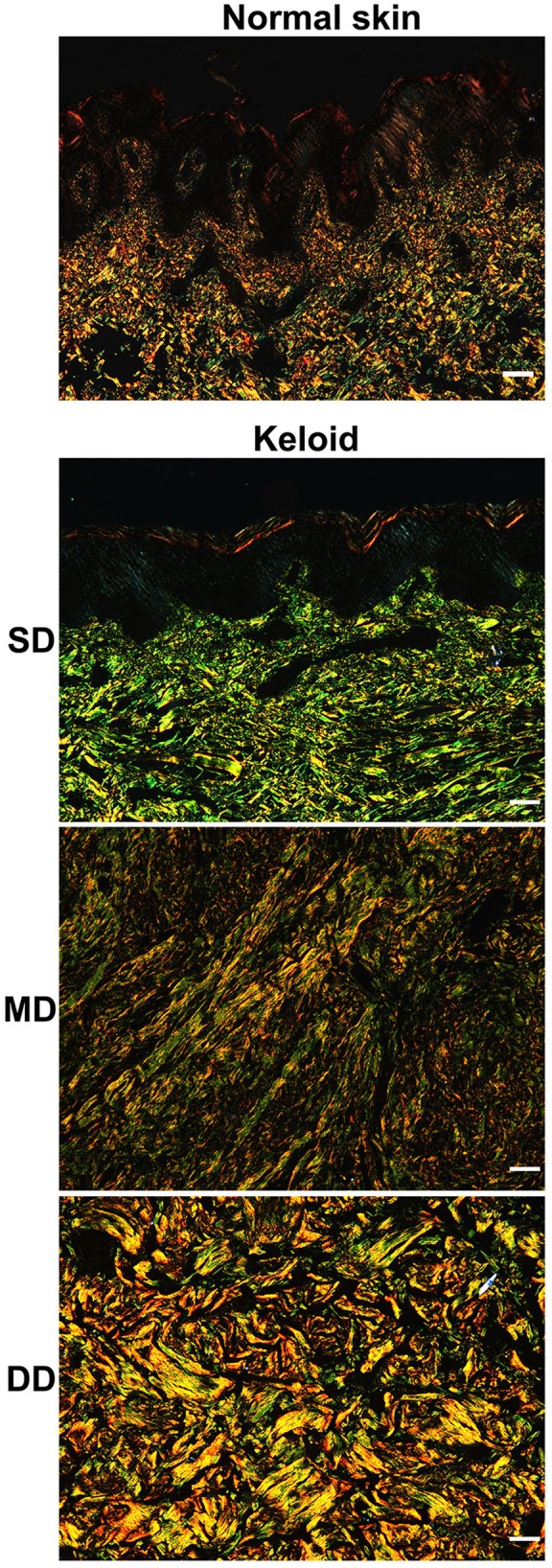
Collagen composition in keloid lesions and normal skin. The type of collagen composition was determined by sirius red staining. Scale bar = 100 μm. SD, superficial dermis; MD, middle dermis; DD, deep dermis.

To determine the type of collagen in keloid lesions and normal skin, Sirius Red staining was performed. In the dermis of the normal skin, type I collagen fibers (yellow) were the main component of ECM, while type III (green) comprised only a small portion. Also, the composition of collagen fibers did not display differences at different depths in normal skin. Consistent with the results of the H&E staining, type I and III collagen fibers were fine, as well as loosely and irregularly arranged in the normal dermis (Figure 4).

Compared with the normal skin, collagen fibers were thicker in the dermis of the keloid and the composition was different at different depths (Figure 4). In the SD of the keloid, the amount of type III collagen fibers was significantly greater than type I collagen fibers. Type III collagen fibers progressively decreased in the MD of the keloid, whereas type I collagen increased. Type I collagen fibers comprised the majority of the ECM in the DD of the keloid. Also, parallel arrangement of type I and III collagen fibers was found in the SD and MD of keloid, but in the DD of keloid, collagen fibers were irregularly arranged.

### Fibroblasts migration in keloid dermis at different depths

**Figure 5:**
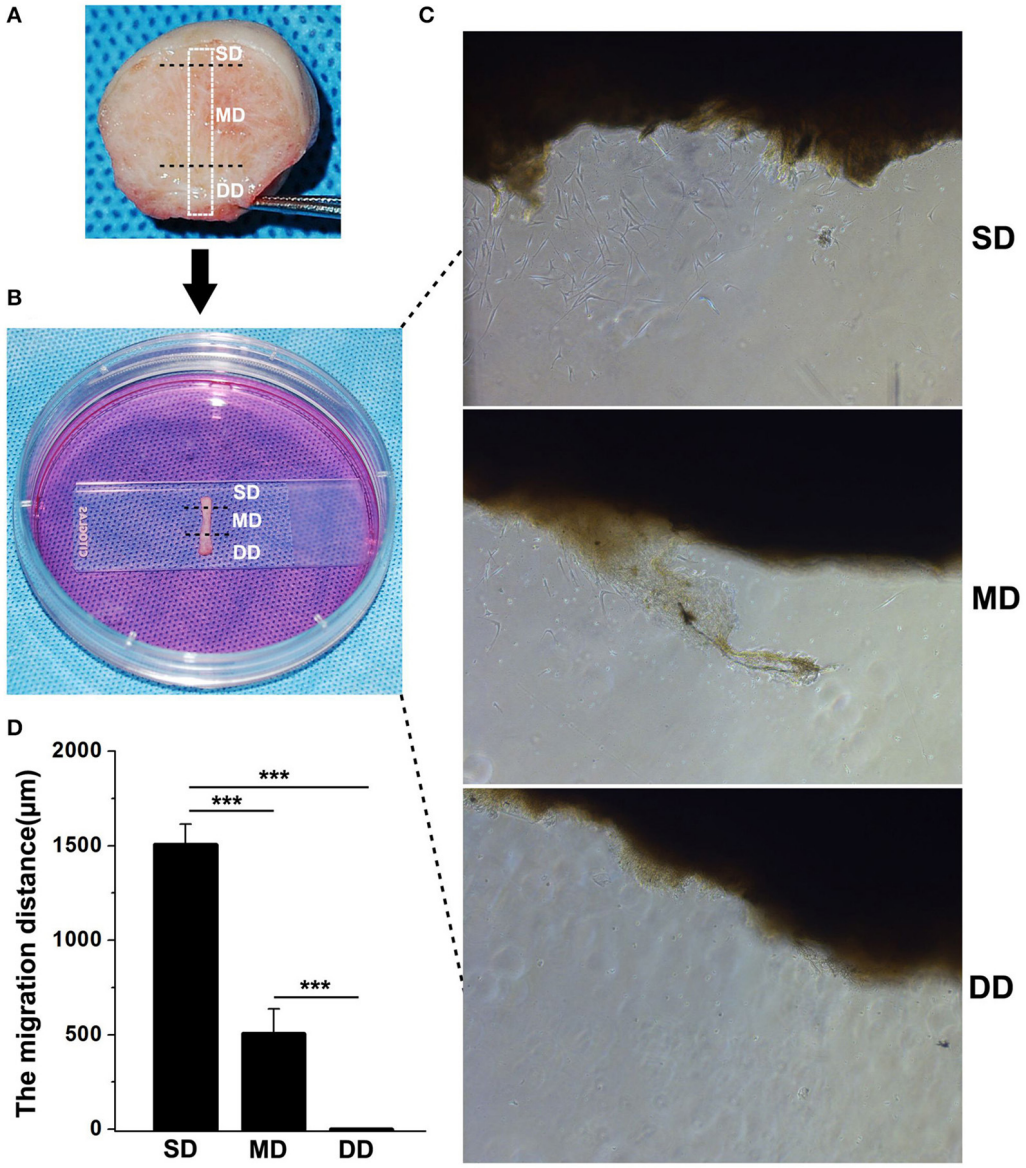
Fibroblasts migration in keloid dermis at different depths. **(A)** Tissue strip containing full-thickness keloid dermis was cut out. **(B)** Tissue strip was fixed by glass and cultured in medium. **(C)** Fibroblast migrating was observed after 7 days and **(D)** The migration distance was measured. SD, superficial dermis; MD, middle dermis; DD, deep dermis. ^***^Behavior, *p* < 0.001.

The migration of fibroblasts from keloid dermis at different depths was investigated using tissue culture (Figure 5). Seven days after incubation, fibroblasts had migrated from the SD of keloid tissue. There were a few fibroblasts observed migrating from the MD of the keloid. However, there were no fibroblasts migrating from the DD of the keloid. The migration distance was measured and results showed fibroblasts from SD migrated fastest.

## Discussion

In the present study, different histological features were identified at different depths in the keloid dermis, which may explain the conflicting findings from different studies. Taking collagen fibers as an example, some studies have reported that collagen fibers are haphazardly oriented in keloid lesions (Santucci et al., 2001), but others have reported collagen fibers to be organized in a more parallel manner (Verhaegen et al., 2009). Moreover, some studies have reported the type I/III collagen ratio is increased in keloids (Weber et al., 1978; Hayakawa et al., 1979), while other studies have suggested that the ratio is decreased (Friedman et al., 1993). As this study has demonstrated a different orientation and type of collagen in different depths of the keloid dermis, contradictory conclusions may be due to the different depths of the biopsies of the keloids that were performed.

A number of studies have indicated an important role for lymphocytes in the development of fibrosis. In a study we previously reported, we showed that B-lymphocytes and T-lymphocytes were present in keloid dermis (Jiao et al., 2015). In the present study, the infiltration of T-lymphocytes was found to be perivascular in the SD of the keloid. It has been reported that perivascular T-lymphocyte infiltration, consisting mainly of activated T-lymphocytes, is found in the skin of patients with systemic sclerosis (Kalogerou et al., 2005), which is also a fibrotic skin disease. T-lymphocytes are believed to promote fibrosis by secreting cytokines such as IL-4, IL-5, IL-6, IL-10, and IL-13 to stimulate the synthesis of collagen by human fibroblasts (Wynn, 2004). B-lymphocytes could directly or indirectly induce tissue fibrosis by local effects, such as antigen presentation, cytokine release, T cell differentiation, dendritic cell modulation, and/or cell-cell contact, or by long-range effects via antibodies (Lipsky, 2001; Marra et al., 2009). For example, B-lymphocytes produce cytokines IL-4, IL-6, IL-13 and TGF-β1 directly promoting a fibrosis response (Mosmann, 2000; Hasegawa et al., 2005). Additionally, the cell-cell interaction has been observed between the fibroblasts and lymphocytes in keloid (Shaker et al., 2011), indicating lymphocytes may promote fibroblasts to synthesize collagen through direct gap junctions.

The predominant collagen types in the human dermis are types I and III, which account for >95% of the bulk of all of the collagen, with approximately 85% being type I collagen (Uitto et al., 1985). At the beginning of wound healing, fibroblasts synthesize type III collagen, which happens as early as 2 or 3 days following wounding. As wound healing progresses, the synthesis of type III collagen is decreased gradually, accompanied by a type I collagen increase at 6 or 7 days following wounding (Diegelmann et al., 1975; Hayakawa et al., 1979). In the current study, type III collagen accounted for the majority of ECM in the superficial tissue layer of the keloid, and the infiltration of lymphocytes and activated fibroblasts were also found in this layer. From the superficial to deep layers of the keloid dermis, the number of fibroblasts decreased and the fibroblasts were transformed from an active state to a static state, and the type I /III collagen ratio and the amount of hyalinized collagen increased.

One of the key features of keloids is the progressive invasion into the adjacent normal skin, exceeding the original wound margin. Migration is a cellular behavior commonly observed during wound healing and metastasis, where fibroblasts or cancer cells undergo dynamic responses featured by rapid focal adhesion turnovers, actin polymerizations and traction force generations (Footer et al., 2007; Harn et al., 2015). Research has indicated that fibroblasts from keloid dermis show a higher migration pattern in comparison with fibroblasts from normal skin or hypertrophic scar (Lim et al., 2006; Song et al., 2012; Yun et al., 2015). In the present study, we also showed that fibroblasts from the superficial dermis of keloid migrate more rapidly when compared with fibroblasts from the middle or deep dermis.

Our results suggest the SD may initiate keloid formation. In the SD, lymphocytes may produce certain cytokines in a paracrine manner and increase the proliferation of fibroblasts and the synthesis of collagen. With the deposition of ECM, fibroblasts are buried in the ECM, which reduces the effects of lymphocytes, and these fibroblasts produce more type I instead of type III collagen. In the deep layer of the keloid dermis, the disappearance of fibroblasts may be due to apoptosis or some other mechanism, decrease in ECM synthesis results in degeneration of collagen fibers, which may explain why few cells and hyalinized collagen fibers were found in the DD of keloid lesions.

Because the SD is important for keloid formation and the infiltration of lymphocytes is increased in the margin of keloids, we would suggest that intralesional injection and other treatments should be performed in the superficial layer of the keloid dermis, especially in the margin of keloids. Because the superficial layer is easily accessed, the development of some medicine for external use feasible. Also, this study has shown that pathological changes were found in the surrounding normal skin, where treatment should also be performed. Our study provides a better understanding of the histological features of the keloid dermis to help guide such local therapeutic strategies.

## Author contributions

HJ conceived and performed the experiments, collected and analyzed the data, and drafted the manuscript. TZ assisted with the collection of data. JF helped in harvesting the keloids and normal skin specimens. RX conceived and designed the project, oversaw the collection of results and data interpretation, wrote the manuscript and had primary responsibility for final content. All authors read and approved the final manuscript.

### Conflict of interest statement

The authors declare that the research was conducted in the absence of any commercial or financial relationships that could be construed as a potential conflict of interest.
